# P33 TB or not TB... That is the question!

**DOI:** 10.1093/rap/rkae117.064

**Published:** 2024-11-05

**Authors:** Sana Sharrack, Caroline Zollinger-Read

**Affiliations:** Department of Rheumatology, Sheffield Teaching Hospitals, NHS Foundation Trust, Sheffield, United Kingdom; Department of Rheumatology, Sheffield Teaching Hospitals, NHS Foundation Trust, Sheffield, United Kingdom

## Abstract

**Introduction:**

We present the case of a 75-year-old male with no risk factors for latent tuberculosis (TB), who was diagnosed with rheumatoid arthritis and appropriately escalated to adalimumab after failing two csDMARDs. His pre-biologic screen revealed a negative IGRA and normal chest X-Ray. He responded well but then developed biopsy-proven giant cell arteritis (GCA), and after commencing additional high dose prednisolone, developed systemic upset with a repeat chest X-Ray revealing miliary TB. This case highlights the possibility of falsely negative IGRAs in patients already on csDMARDs and raises the question of whether we should screen for latent TB in csDMARD-naïve patients.

**Case description:**

We present the case of a 75-year-old Caucasian male with no known past medical history who presented to rheumatology and was diagnosed with a classic presentation of seropositive rheumatoid arthritis. He was treated initially with methotrexate but this did not control his disease adequately, and subsequently sulfasalazine was added. Unfortunately he could not tolerate high doses of sulfasalazine and hydroxychloroquine was introduced, which again he could not tolerate. At this point, he was discussed in the local departmental MDT and a decision was made to commence adalimumab. A pre-biologic screen at this point revealed a normal chest X-Ray and a negative QuantiFERON TB. He had no prior history of TB and no known risk factors for latent TB. He was in remission at 6 months and sulfasalazine was stopped. Unfortunately 6 months later, he developed diverticulitis and then headaches typical for GCA with raised inflammatory markers and biopsy-confirmed temporal arteritis. He was commenced on high dose prednisolone in addition to continuation of adalimumab and methotrexate. Shortly afterwards, he developed worsening abdominal pain, loose stool, fevers and a CT of the abdomen and pelvis revealed extensive diverticular disease but no diverticulitis. Incidentally, it picked up tree-in-bud changes in the lower lungs in keeping with possible chest infection, treated with two weeks of levofloxacin. He re-presented two weeks later with ongoing fever, rigors and systemic upset and a chest X-Ray showed evidence of miliary TB. His adalimumab and methotrexate were held, and TB treatment was commenced. He was on 17.5 mg of oral prednisolone, which was incrementally increased to 80 mg in light of rifampicin effect. Subsequently, despite high dose prednisolone, our patient developed a leukocytoclastic vasculitic (LCV) rash thought to be related to rifampicin. The rifampicin was continued due to TB disease burden and the rash resolved after 10 days.

**Discussion:**

Our patient presented with a typical presentation of rheumatoid arthritis including inflammatory arthralgia, positive serology (RF and CCP) and raised acute phase reactants (CRP and ESR) which was consistent with a diagnosis of rheumatoid arthritis as per ACR-EULAR classification. Treatment was escalated appropriately to bDMARDs with pre-immunosuppression screen. The current EULAR and BSR guidelines do not advise pre-csDMARD TB screening, although EULAR does identify that there are cases of TB reactivation with csDMARD and high doses of steroids. In our case, it is unclear if our patient had previously latent TB with a falsely negative QuantiFERON, or whether he newly acquired TB whilst immunosuppressed on adalimumab that progressed to miliary TB with addition of high dose steroids. LCV has rarely been reported in association with TB. There are two types of TB-related vasculitis described in the literature; a hypersensitivity-induced reaction due to the circulating immune complexes in pulmonary TB, leading to deposition in the vascular wall and consequently LCV, and a second type associated with the initiation of anti-TB medication, particularly with rifampicin. Anti TB medication-associated vasculitis typically improves upon withdrawal of the medication. Rifampicin is known to induce hepatic drug metabolising enzymes and will increase the plasma clearance of prednisolone by 45% and reduce the bioavailability of the drug to the tissues by 66%. Therefore, the effectiveness of prednisolone is considerably reduced when co-prescribed with rifampicin. This case highlights the importance of considering higher than normal doses of prednisolone when patients are on TB treatment, to compensate for increased metabolism. Our case demonstrates that patients with rheumatoid arthritis can get GCA despite being on immunosuppression, although this is rarely reported in the literature.

**Key learning points:**

• We have learnt from this case that IGRA can be falsely negative in patients with latent TB who are already established on immunosuppression, so this may be falsely reassuring. Our local TB Quality Improvement Project (QIP) work has identified 8 cases of positive/latent TB by screening pre-csDMARD compared to 0 for patients established on csDMARDs. Our patient interestingly had no risk factors for TB including being Caucasian, no previous TB exposure and no travel to high incidence countries. There are no international standardised guidelines on screening for latent TB in DMARD-naive patients. This case demonstrates the need to do a thorough risk screen for TB, with a careful medical history, clinical examination and tests such as QuantiFERON and chest X-ray in not only pre-bDMARD patients but also pre-csDMARD patients. LCV is an unusual and challenging presentation in the context of TB. It can be TB-related vasculitis or rifampicin-associated LCV. Often there is reluctance to stop rifampicin due to concern about TB disease burden, thereby discussion with specialities such as infectious diseases and dermatology is paramount to weigh up the risks of continuing TB treatment versus stopping. Although rarely seen, TB itself should be considered as an important cause of LCV. Lastly, this case report represents a case of GCA occurring in a patient already on adalimumab and methotrexate (a steroid-sparing agent for GCA). Although rare, clinicians should be vigilant in considering a diagnosis of GCA in patients with established immunosuppression and should appropriately investigate and treat. This case is complex, demonstrating an unusual presentation of GCA in the context of a patient already on immunosuppression, who subsequently developed military TB despite a negative pre-immunosuppression screen and no risk factors for TB. It contributes to the ongoing discussion about the need for TB screening in all DMARD-naive patients.

